# A Handbook of Materia Medica, Pharmacy and Therapeutics

**Published:** 1903-02

**Authors:** 


A HANDBOOK OF MATERIA MEDICA, PHARMACY AND THERAPEUTICS. Including the Physiological Action of Drugs, the Special Therapeutics of Disease, Official and Practical Pharmacy, and minute directions for prescription writing. By SAMUEL O. L. POTTER, A M., M. D., M. R. C. P., Lond., formerly Professor of the Principles and Practice of Medicine in the Cooper Medical College of San Francisco. Ninth edition, revised and enlarged. Octavo, pp. 951. Philadelphia: P. Blakiston's Son & Co. 1902. [Price, $5 00 net.]

This is hardly a handbook for it is an octavo of 951 pages. It is as complete a study of drugs as one can wish. No new remedy of proved value is neglected. The book is arranged in three parts. Part I. is an alphabetical list of drugs. Each drug is described, physiological action and therapeutic indications being given. The animal extracts mentioned are the thyroid, bone-marrow, nuclein, orchitic, cerebrin, adrenalin, splenic, lymphatic, pancreatic, parotid, ovarian, uterine and mammary gland. In regard to these Potter says, "Most of them are yet on trial and the limits of their utility in medicine are by no means defined."

Part II. is devoted to pharmaceutical preparations and directions for prescription writing.

Part III. is devoted to special therapeutics. The leading authorities are freely quoted and many valuable suggestions are given for almost all the ills that human flesh is heir to.

We believe the work will always be a favorite one with physicians as it is written with care and is fully up to date.

